# Perceptions of Zika Virus Risk during 2016 Outbreak, Miami-Dade County, Florida, USA

**DOI:** 10.3201/eid2407.171650

**Published:** 2018-07

**Authors:** Imelda K. Moise, Joseph Kangmennaang, Tricia Caroline S.G. Hutchings, Ira M. Sheskin, Douglas O. Fuller

**Affiliations:** University of Miami, Coral Gables, Florida, USA (I.K. Moise, T.C.S.G. Hutchings, I.M. Sheskin, D.O. Fuller);; University of Waterloo, Waterloo, Ontario, Canada (J. Kangmennaang)

**Keywords:** Zika virus, health belief model, knowledge, attitudes, practices, KAP, Miami-Dade County, viruses, vector-borne infections, perceptions, United States, Florida

## Abstract

We conducted a survey on Zika virus perceptions and behaviors during the 2016 outbreak in Miami-Dade County, Florida, USA. Among women, Zika knowledge was associated with having a bachelor’s degree. Among men, knowledge was associated with knowing someone at risk. Interventions during future outbreaks could be targeted by sex and education level.

Misconceptions about arboviruses transmitted by *Aedes* spp. mosquitoes, such as Zika virus, can lead to misplaced reactions and affect local public health officials’ abilities to contain outbreaks (*1*–*3*). Despite media campaigns on Zika virus, misperceptions persisted during the 2016 outbreak among some subgroups in Miami, Florida, USA (*4*). More than 4 in 10 Americans mistakenly thought that Zika virus infection was fatal and that symptoms were noticeable (*5*).

We conducted a structured bilingual (English, Spanish) telephone survey with a random sample of adults in late spring (May 1–June 30, 2016), when the Zika virus outbreak began in Florida. We applied the basic concepts of the Health Belief Model (HBM) in an attempt to understand perceptions of Zika virus risk and prevention practices in Miami-Dade County, Florida, the epicenter of the 2016 Zika virus outbreak (*6*).

The HBM provided the framework enabling effective structuring of messages to influence behavioral change in the context of health communication strategies for Zika virus prevention and control. According to the HBM, persons are influenced by their perceived susceptibility to a disease and the severity of that disease (*7*). To use the HBM, participants must have the ability to implement a desired behavior, self-efficacy (i.e., confidence in their ability to implement that action), and cues to action (which could lead to health behavior changes) (*7*). Because Zika virus infection mainly affects pregnant women (*8*,*9*), we report differences in perception and behavior by sex. Our target sample size was 421, with a power of 0.90 and margin of error of 0.4. The survey took 10–30 minutes to complete, and ≈62% (262/421) of the target population participated. 

We determined predictive factors of Zika virus knowledge (dependent variable, values 0 or 1) by using multivariate logistic regression with a log-link function adjusted for demographics (age, sex, employment status, education level, income level) and all other variables of the HBM. We presented data as adjusted odds ratios (aORs) with 95% CIs. A low score (0–7 points) on the Zika virus knowledge test indicates the participant correctly answered 0–7 questions and suggests the respondent had simply heard of Zika and knew that mosquitoes could transmit Zika virus. A high score (8–12 points) indicates the participant correctly answered 8–12 questions and suggests the respondent had a good understanding of microcephaly and Guillain-Barré syndrome.

Of the 262 survey participants, 149 (56.9%) were women and 113 (43.1%) were men; age range was 18–94 (mean 49, SD 19) years. More than half (56.9%) of participants were foreign born, 185 (70.6%) considered themselves Hispanic or Latino, and 138 (52.7%) were married. More women (36.9%) than men (31.0%) scored high (8–12 points) for Zika virus knowledge (Technical Appendix Table).

A total of 53.0% of women and 49.6% of men felt somewhat confident they could protect their households from contracting Zika (Technical Appendix). Personal protective measures included window and door screens, checking for and draining standing water, and using repellents. A higher percentage of women (53.7%) than men (42.5%) perceived Zika to be a severe disease, and women (50.4%) were more likely than men (43.6%) to report fear of contracting Zika.

Taking action to protect oneself against Zika virus infection (aOR 2.39, p = 0.01) and knowing someone pregnant (cue to action) (aOR 2.13, p = 0.10) were associated with a higher knowledge of Zika virus (Table). This high level of knowledge might be attributable to the Florida Department of Health’s aggressive information campaign and a Zika virus information hotline created to help inform the public about Zika virus and procedures to avoid infection. Participants with bachelor’s degrees (aOR 2.37, p = 0.01) were also more likely to be knowledgeable about Zika virus than those without bachelor’s degrees.

**Table:** Multivariate logistic regression analysis of variables associated with high Zika virus knowledge among Miami-Dade County residents, by sex, Florida, USA, 2016*

Category	aOR (95% CI)		
	Total, n = 262	Female, n = 149	Male, n = 113
Constant†	0.07 (0.01–0.37)‡	0.05 (0.004–0.647)§	0.066 (0.004–1.094)¶
Self-efficacy			
Confidence to protect household from Zika virus infection			
Medium	1.29 (0.59–2.77)	1.36 (0.45–4.12)	1.97 (0.50–7.68)
High	1.26 (0.52–3.05)	1.15 (0.32–4.13)	2.81 (0.59–13.14)
Took action to protect against Zika virus			
No	Referent	Referent	Referent
Yes	2.39 (1.24–4.61)‡	2.30 (0.882–5.999)¶	3.18 (1.07–9.44)§
Severity of disease			
Severity of Zika virus infection			
Less severe	Referent	Referent	Referent
Somewhat severe	1.09 (0.38–3.16)	1.24 (0.27–5.67)	0.84 (0.13–5.38)
Very severe	1.35 (0.46–3.96)	2.62 (0.61–11.08)	0.53 (0.061–4.54)
Severity of microcephaly			
Not severe	Referent	Referent	Referent
Somewhat severe	1.07 (0.51–2.27)	1.26 (0.45–3.58)	1.04 (0.31–3.51)
Very severe	0.79 (0.34–1.87)	1.07 (0.32–3.58)	0.52 (0.12–2.12)
Susceptibility to disease			
How likely are you to contract Zika virus			
Very unlikely	Referent	Referent	Referent
Somewhat unlikely	1.56 (0.82–2.96)	1.34 (0.557–3.226)	2.45 (0.83–7.26)
Likely	2.36 (0.896–6.25)¶	1.36 (0.323–5.795)	3.21 (0.70–14.63)
Benefits of action			
Taking action against Zika virus			
Beneficial	Referent		
Not beneficial	−0.91 (−2.55 to 0.73)	NA	NA
Possible cues to action			
Knowing someone at risk for Zika disease (pregnant or planning on being pregnant)			
No	Referent	Referent	Referent
Yes	2.13 (0.95–4.77)¶	1.15 (0.41–3.22)	11.73 (2.28–60.28)‡
Demographics			
Age	0.99 (0.97–1.01)	0.99 (0.96–1.02)	1.00 (0.97–1.04)
Sex			
M	Referent		
F	1.18 (0.63–2.20)	NA	NA
Employment status			
Not in the workforce	Referent	Referent	Referent
In the work force	1.23 (0.579–2.605)	1.02 (0.35–2.97)	1.15 (0.33–4.02)
Education level			
Less than bachelors	Referent	Referent	Referent
Bachelor’s degree or higher	2.37 (1.25–4.47)‡	2.92 (1.199–7.12)§	1.54 (0.53–4.42)
Income level			
<$50,000	Referent	Referent	Referent
$50,000-$100,000	0.98 (0.46–2.09)	1.15 (0.44–2.98)	0.65 (0.18–2.25)
>$100,000	2.06 (0.88–4.78)¶	2.51 (0.72–8.73)	1.75 (0.42–7.32)
Don’t know or NA	0.86 (0.33–2.21)	1.73 (0.52–5.78)	0.04 (0.006–0.304)‡

*aOR, adjusted odds ratio; NA, not applicable. †The constant is the expected mean value of *y* when *x* equals zero. ‡p<0.01. §p<0.05. ¶p<0.10.

Among women, Zika virus knowledge was higher among those who had taken action to prevent Zika virus infection (aOR 2.30, p = 0.10) and those with bachelor’s degrees (aOR 2.92, p = 0.05). However, among men, Zika virus knowledge was higher among those who knew someone at risk for Zika (aOR 11.73, p = 0.01) and those who took action to prevent Zika virus infection (aOR 3.18, p = 0.05).

Our analysis indicates that women were more concerned about Zika than were men in Miami-Dade County and that those with bachelor’s degrees were more knowledgeable than were those without. Therefore, targeting prevention and treatment interventions by sex and education level should be considered to maximize positive outcomes in high-risk areas during outbreaks (*10*). For local governments, planning and implementing effective interventions aimed at preventing and controlling mosquitoborne disease outbreaks require ongoing assessments of knowledge, attitudes, and practices that are sensitive to local residents’ health practices and concerns. These findings have critical implications for future studies that seek more accurate and confirmatory evidence on the association between socio-demographics and Zika virus–related health practices.

Technical Appendix Characteristics of Miami-Dade County residents, by sex, Florida, USA, 2016.
