# Supplementary material for: Perceptions of Zika Virus Risk during 2016 Outbreak, Miami-Dade County, Florida, USA
Source: Emerg Infect Dis. 2018 Jul;24(7):1379–81. doi: 10.3201/eid2407.171650 (PMC6038743; doi:10.3201/eid2407.171650)
Supplement: Technical Appendix — Characteristics of Miami-Dade County residents, by sex, Florida, USA, 2016. [file 17-1650-Techapp-s1.pdf]

Article DOI: https://doi.org/10.3201/eid2407.171650

Perceptions of Zika Virus Risk during 2016 Outbreak, Miami-Dade County, Florida, USA

Technical Appendix

Technical Appendix Table. Characteristics of Miami-Dade County residents, by sex, Florida, USA, 2016*

Variable	Male, n = 113, %	Female, n = 149, %	Pearson χ^2	p value
Zika virus knowledge, dependent variable			1.01	0.32
Low, 0–7	69.03	63.09		
High, 8–12	30.97	36.91		
Confident can protect household from Zika virus infection			1.26	0.53
Little or not confident	27.43	21.48		
Somewhat	49.56	53.02		
Very	23.01	25.50		
Take action to protect oneself			1.42	0.23
No	40.71	33.56		
Yes	59.90	66.44		
Perceived severity of Zika virus infection			4.81,	0.09
Little or no	7.08	9.40		
Somewhat	50.44	36.91		
Very	42.48	53.69		
Perceived severity of microcephaly			5.71	<0.05
Little or no severity	29.20	18.24		
Somewhat	41.59	54.73		
Very	29.20	27.03		
Likely to contract Zika virus infection			6.15	<0.04
Very unlikely	50.44	43.62		
Somewhat unlikely	30.97	44.97		
Very likely	18.58	11.41		
Benefits of taking action to prevent Zika virus infection			2.94	0.08
No	38.94	28.86		
Yes	61.06	71.14		
Cues to action (know someone who is pregnant)			1.28	0.25
No	89.38	484.56		
Yes	10.62	15.44		
Employment status			3.32	0.06
In work force	74.34	63.76		
Not in workforce	25.66	36.24		
Education			0.17	0.67
Less than bachelors	49.56	46.98		
Bachelors or higher	50.44	53.02		
Gross income level, USD			17.70	<0.001
<$50,000	26.55	47.65		
$50,000-$100,000	33.63	23.49		
>$100,000	28.32	13.42		
Don’t know	11.50	15.44		

*USD, US dollar.
